# Correction of cubitus valgus and reconstruction of lateral humerus condylar defect using tricortical iliac graft in pediatric patients

**DOI:** 10.1051/sicotj/2023028

**Published:** 2023-11-30

**Authors:** Mohamed Hussein Fadel, Mohamed Hassan Hashem, Ahmed Ramy

**Affiliations:** Department of Orthopedic Surgery, Faculty of Medicine, Helwan University Cairo Egypt

**Keywords:** Lateral condyle, Nonunion, Tricortical graft, Cubitus valgus, Reconstruction

## Abstract

*Background*: Neglected non-united lateral humeral condyle fractures in pediatrics are a probable cause of cubitus valgus deformity which is a disabling complication. The ideal management for this condition is still debatable. This study aimed to evaluate the reconstruction of a non-united lateral humerus condylar fracture complicated by cubitus valgus using a tricortical iliac crest graft in pediatric patients. *Patients and methods*: Twenty children suffering from cubitus valgus as a complication after a non-united fracture of the lateral humeral condyle were included in this study. They were managed by open reduction, screw fixation, and reconstruction by an autologous tricortical iliac bone graft. We compared the preoperative and postoperative range of motion of the elbow, alignment, and elbow function using the Mayo elbow performance index. *Results*: There was a statistically significant improvement in the elbow range of motion postoperatively, and there was a highly significant improvement regarding the elbow alignment and function. *Conclusion*: Open reduction, screw fixation, and reconstruction by the autologous tricortical iliac bone graft is an effective technique for the management of cubitus valgus due to neglected non-united lateral humeral condyle fractures in pediatrics.

## Introduction

Fractures around the elbow are the most common fractures in pediatric populations, with an incidence of about 28.4% of all pediatric fractures. These fractures carry the risk of several disabling complications, including neurovascular injuries, cubitus valgus/varus deformities, Volkmann’s contractures, nonunion, and ulnar nerve palsies [1].

Humeral lateral condyle fractures are known for nonunion. If early proper management is not achieved, they can lead to a cubitus valgus malalignment. In neglected or mismanaged cases with deformity and limited range of motion, surgical management is challenging, and a well-tailored preoperative plan is required [2].

Lately presented fracture of the lateral condyle humerus in pediatrics is a surgical dilemma. The decision of fixation or corrective osteotomies and ulnar nerve transposition or sometimes both procedures combined is a controversial topic [3].

The incidence of satisfactory outcomes measured by functional elbow scores decreases with the increase of the time from injury. A satisfactory result can be anticipated in the first 5 weeks, this declines toward the 12th week, and afterward, the results significantly deteriorate [4].

The aim of an effective management plan includes anatomical reduction – especially of the articular surface – and rigid fixation allowing early motion. One of the major difficulties a treating surgeon faces in reconstruction is a proper reduction of the lateral condyle, especially when there is major bone loss. These challenging bone voids can be managed using a tricortical iliac crest graft to achieve an appropriate reconstruction of the lateral condyle [5].

Several treatment procedures have been described such as distal humerus osteotomy (dome osteotomy, varus osteotomy, or open wedge Milch’s displacement osteotomy), or to go for open reduction, filling the defect with bone graft, and fixation either by screws or K-wires [6].

For the time being, few studies have addressed the use of autologous iliac bone grafts for the management of neglected lateral humeral condyle in pediatrics.

### Aim of the work

This study aims to evaluate the reconstruction of a non-united lateral humerus condylar fracture complicated by cubitus valgus using a tricortical iliac crest graft in pediatric patients, regarding restoring elbow alignment, range of motion, and function.

## Patients and methods

In the period from August 2021 and April 2023, a prospective case study was conducted including 20 cases (7 females and 13 males). Patients’ ages had a mean of 7.85 (6–11) years. Patients were referred from the outpatient clinic of the orthopedic surgery department, suffering from cubitus valgus as a complication of a non-united fracture of the lateral humeral condyle. The mean interval between the lateral condyle fracture and the presentation was 22 (18–29) weeks.

This study was approved by the Ethics Committee of the Department of Orthopedic Surgery, Faculty of Medicine, Helwan University. Informed consent was taken from each patient’s parent or legal guardian after explaining the steps of the procedure, its anticipated benefits, potential complications, and follow-up regimen.

The comparison between preoperative and postoperative data was done by using a Paired *t*-test. The *p*-value was considered significant as the following: *p* > 0.05 = non-significant, *p* < 0.05 = significant, *p* < 0.001 = highly significant (HS).

Demographic data are listed in Table 1, and radio-clinical data are listed in Table 2.

**Table 1:** Demographic data.

No	Sex	Age in years	Duration from the fracture in weeks	Duration of follow-up in months	Side
1	Female	8	28	6	Left
2	Female	7	26	5	Right
3	Female	9	24	7	Right
4	Male	7	26	5	Right
5	Male	8	24	8	Right
6	Female	6	22	6	Right
7	Male	8	18	5	Right
8	Male	6	26	5	Right
9	Male	6	22	7	Right
10	Female	7	20	4	Right
11	Male	9	22	6	Right
12	Male	11	20	4	Left
13	Male	8	18	8	Right
14	Female	6	18	9	Right
15	Male	8	20	5	Right
16	Female	7	22	6	Right
17	Male	9	18	7	Right
18	Male	10	18	8	Left
19	Male	10	20	4	Left
20	Male	7	22	7	Right

**Table 2:** Radio-clinical data.

Number	Preoperative humeroulnar angle	Postoperative humeroulnar angle	Preoperative elbow range in degrees	Final follow-up range	Preoperative Bauman angle in degrees	Postoperative Bauman angle in degrees	Preoperative MEPI	postoperative MEPI
1	40	10	0–90	0–120	60	80	40	80
2	35	12	0–110	0–110	65	75	55	75
3	30	8	0–110	0–120	65	78	55	80
4	40	10	0–100	0–120	60	75	45	80
5	30	7	0–110	0–125	68	78	55	70
6	20	8	0–115	0–130	68	75	55	80
7	25	10	0–105	0–135	65	70	55	80
8	40	12	0–80	0–90	65	70	40	55
9	35	10	0–110	0–120	65	75	60	80
10	35	10	0–110	0–120	65	70	55	80
11	40	12	0–100	0–125	60	78	40	75
12	20	8	0–110	0–120	68	76	55	80
13	20	6	0–95	0–110	68	78	55	85
14	25	5	0–100	0–125	68	78	50	80
15	20	5	0–110	0–120	64	74	55	80
16	40	12	0–90	0–110	65	78	40	70
17	30	10	0–100	0–120	68	68	55	80
18	20	10	0–105	0–120	68	80	50	85
19	25	5	0–110	0–125	62	74	55	80
20	30	10	0–100	0–120	64	70	55	85

Inclusion criteria include patients with cubitus valgus due to non-united fracture of the lateral humeral condyle, both sexes with age less than 12 years while exclusion criteria include cubitus valgus due to other causes, fractures not needing grafts, previous elbow operations, and cubitus valgus with infected nonunion and unhealthy skin coverage.

A proper preoperative evaluation was performed for all patients, this included history taking, examination, and radiographs. The examination included assessment and recording of the neurovascular status and soft tissue evaluation (Figures 1 and 2).

**Figure 1:**
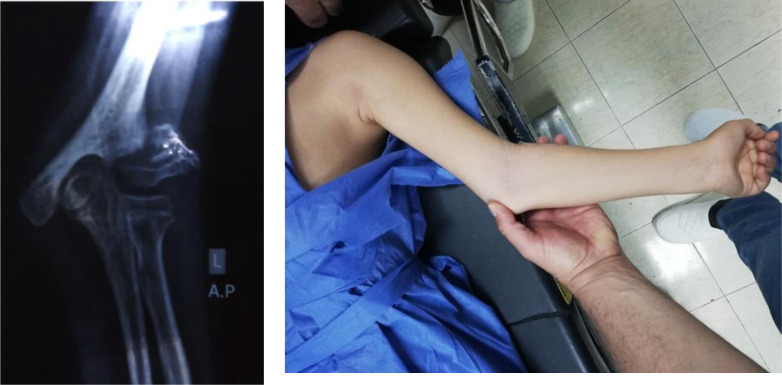
Preoperative X-ray and clinical photo of case no. (1).

**Figure 2:**
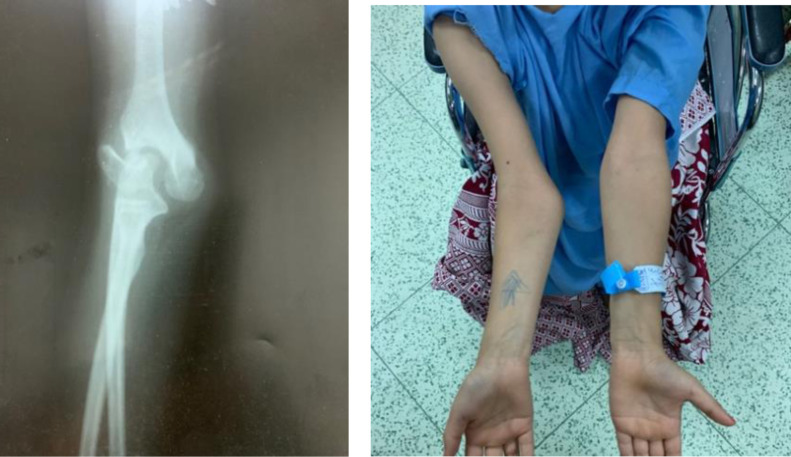
Preoperative X-ray and clinical photo of case no. (3).

The elbow function was scored using the Mayo elbow performance index (MEPI) (Table 3) [6].

**Table 3:** The Mayo elbow performance index [7].

Measured item	Findings	Score
Pain	No pain	45
	Mild	30
	Moderate	15
	Severe	0
Elbow range of motion	>100°	20
	50–100°	15
	<50°	5
Stability	Stable	10
	Moderately unstable	5
	Grossly unstable	0
Function	Comb hair	5
	Self-feed	5
	Personal hygiene	5
	Put on shirt	5
	Put on shoes	5

The radiological examination included anteroposterior, lateral, and oblique plain radiographs of the elbow. Comparison radiographs of the contralateral elbow were done to help better understand the geometry of the fracture. These radiographs are used to measure the Baumann’s angle and humeroulnar angle. The size and shape of the tricortical iliac graft were provisionally determined according to the radiographs.

Informed consent was taken from the patient’s parent or legal guardian after thoroughly discussing the steps of the procedure, the possible complications, the anticipated outcomes, and the postoperative rehabilitation regimen.

### Surgical procedure

General anesthesia was used for all patients. One dose of prophylactic antibiotic was given 30 minutes before the incision. Kocher incision was used after the application of a tourniquet. Proper exposure of the defect site was done and the remaining lateral condyle fragment was identified. The proximal fracture site was nibbled to obtain a fresh bleeding surface. A tricortical bone graft was harvested from the ipsilateral iliac crest with the shape and size requirement, and then finely shaped and adjusted to fill the defect between the humeral metaphysis and the remaining fragment.

K-wires were applied to temporarily fix the lateral condyle fragment and the graft to the humerus. The image intensifier was used to check the size and position of the graft and accordingly, any adjustments were made until we achieved the most satisfying reduction and elbow alignment we can achieve.

K-wires were then replaced by 4 mm partially threaded canulated screws for more stable fixation and compression. The wound was closed in layers and a protective above-elbow posterior slab was applied.

### Postoperative care and evaluation

The posterior slab was removed after 3 weeks, and a pouch arm sling was used to allow the patient to start active assisted elbow motion. Serial X-rays were done to assess the union and to make sure that the alignment was preserved. After complete radiological union, the patients were assessed for elbow range of motion and alignment, and MEPI was calculated (Figures 3 and 4).

**Figure 3:**
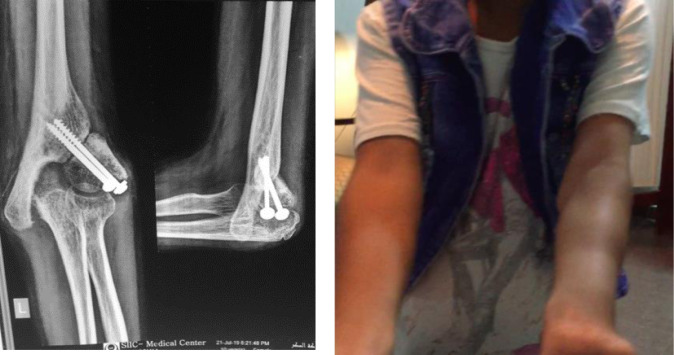
Early postoperative X-ray and 6-month postoperative clinical photo of case no. (1) (left elbow).

**Figure 4:**
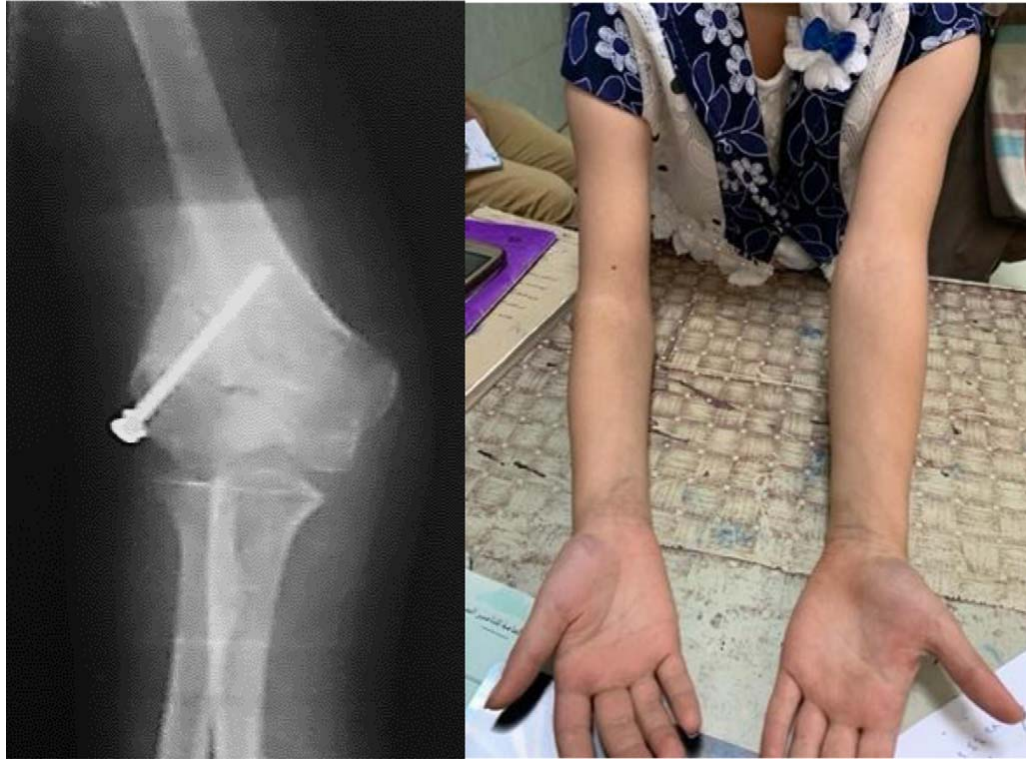
Postoperative X-ray and 7-month postoperative clinical photo of case no. (3) (right elbow).

## Results

Regarding alignment, the average preoperative humeroulnar angle was 30° ± 7.78°, which improved postoperatively to an average of 9° ± 2.38°. There was a highly statistically significant difference between preoperative and postoperative humeroulnar angle. (Fig 5)

**Figure 5:**
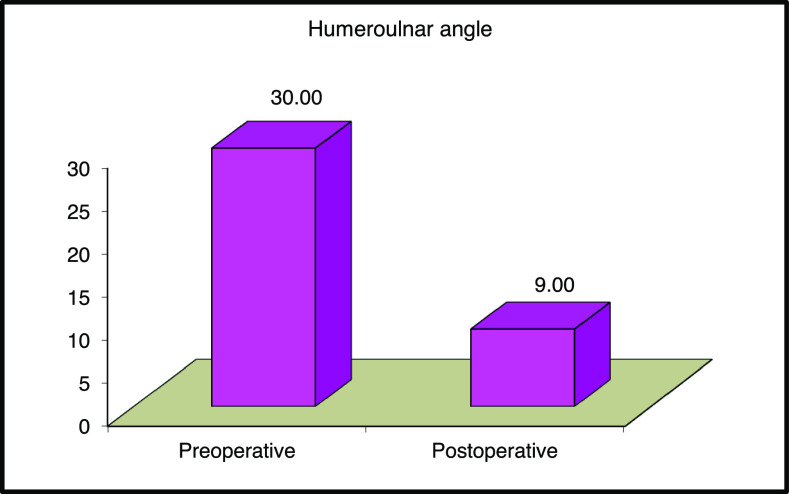
The difference between the average preoperative and postoperative humeroulnar angle.

The average elbow range of motion improved significantly from preoperative 103° ± 8.94° to a postoperative range of 119.25° ± 9.22°. This was calculated as a highly statistically significant difference.

All patients had poor elbow function preoperatively with an MEPI of less than 60. That improved significantly at the last follow-up. The average preoperative MEPI was 51.25 ± 6.46 points, which improved in a highly statistically significant way to reach an average MEPI of 78 ± 6.77 points at the last follow-up. All cases reach full union from 2 to 4 months postoperatively.

Regarding complications, one patient suffered from a superficial wound infection of the iliac graft donner site, he received antibiotics and frequent wound dressings, and the infection resolved with no need for wound debridement.

Another patient suffered from a limited range of motion of the elbow. The child and his parents were not satisfied with the final outcome but his parents decided that they were not going to address the problem any further for the time being.

## Discussion

Cubitus valgus deformity, as a subsequence of lateral humeral condyle nonunion, is one of the most intolerable complications [8]. The valgus deformity that occurs as a complication of nonunion is due to the leaking of synovial fluid into the fracture site, which inhibits fibrin formation, leading to the formation of a secondary callus [9].

The main limitations of this study are the limited number of patients, the relatively short follow-up period, and the absence of comparison with other management options.

The short-term results of this study show significant improvement in postoperative elbow alignment and range of motion, which in return leads to improvement in the overall elbow function which was calculated by the MEPI.

Open reduction and internal fixation for such neglected fractures give the opportunity to refresh the fracture ends, remove any soft tissue obstacles and excessive callus, and fill the residual gap with the proper sized and shaped autograft.

Applying a tricortical bone graft allowed for the restoration of elbow alignment and enhancement of bone healing. Screw fixation – compared to K-wires fixation – allowed for an early beginning of elbow range of motion exercises which may have contributed to the improvement of the final elbow function. None of the patients needed ulnar nerve transposition.

Approximately 50% of all cases of non-unions in pediatrics are around the elbow joint [10]. Fracture of the lateral humeral condyle in children is unfortunately neglected frequently. This could be explained by the difficult clinical and radiological diagnosis [11].

The best management of neglected non-united lateral humeral condyle fracture in pediatrics is still a debatable issue. The less-than-optimal results of operative management hindered its popularity. The risk of avascular necrosis of the lateral condyle, reducing the already limited elbow range of motion, and deterioration of the elbow function are some of the most serious concerns. Still, the best management of lateral humeral condyle fracture is early diagnosis and treatment [12].

Despite that avascular necrosis is a feared complication of open reduction, we luckily did not encounter this problem. We tried to minimize the soft tissue dissection as possible especially at the posterolateral part of the lateral condyle to preserve its blood supply.

One case suffered from a superficial infection at the incision of the iliac crest graft, donor site morbidity is a potential complication of autologous bone graft, but the condition improved after treatment with antibiotics and frequent dressing.

Although most cases had a significant improvement in elbow range of motion, one case did not. We did not find an obvious cause for this as the reduction was accepted, the alignment was restored and there was no evidence of heterotropic ossification. The child’s parents refused to continue physiotherapy or do more investigations and dropped the follow-up after 5 months.

## Conclusion

Open reduction, screw fixation, and reconstruction by the autologous tricortical iliac bone graft is an effective technique for the management of cubitus valgus due to neglected non-united lateral humeral condyle fractures in pediatrics, taking into consideration that precise preoperative planning, careful soft tissue handling, and well-planned postoperative rehabilitation program are of outmost importance to avoid complications and restore elbow function.

## References

[1] Okubo H, Nakasone M, Kinjo M, Onaka K, Futenma C, Kanaya F (2019) Epidemiology of paediatric elbow fractures: a retrospective multi-center study of 488 fractures. J Child Orthop 13(5), 516–521. 31695819 10.1302/1863-2548.13.190043 PMC6808078

[2] Vaish A, Vaishya R, Maini L, Johar A (2020) 3D printing aided elbow deformity correction. Am J Surg Case Rep 2(2), 2–4.

[3] Agarwal A, Qureshi NA, Gupta N, Verma I, Pandey DK (2012) Management of neglected lateral condyle fractures of humerus in children: a retrospective study. Indian J Orthop 46(6), 698–704. 23325975 10.4103/0019-5413.104221 PMC3543890

[4] Shaerf DA, Vanhegan IS, Dattani R (2018) Diagnosis, management and complications of distal humerus lateral condyle fractures in children. Shoulder Elbow 10(2), 114–120. 29560037 10.1177/1758573217701107 PMC5851120

[5] Rathore S, Quadri V, Tapadia S (2016) Reconstruction of lateral humerus condylar defect using tricortical iliac crest graft: a case report. J Med Sci Res 4(2), 72–75.

[6] Johar A, Vaish A, Maini L, Vaishya R (2020) 3D printing aided elbow deformity correction. Am J Surg Case Rep 2674–5046. 10.31487/j.AJSCR.2020.02.05

[7] Longo UG, Franceschi F, Loppini M, Maffulli N, Denaro V (2008) Rating systems for evaluation of the elbow. Br Med Bull 87(1), 131–161. 18539627 10.1093/bmb/ldn023

[8] Skak SV, Olsen SD, Smaabrekke A (2001) Deformity after fracture of the lateral humeral condyle in children. J Pediatr Orthop B 10, 142–152. 11360781

[9] Abed Y, Nour K, Kandil YR, et al. (2018) Triple management of cubitus valgus deformity complicating neglected nonunion of fractures of lateral humeral condyle in children: a case series. SICOTJ, 42, 375–384. 10.1007/s00264-017-3709-6 29214396

[10] Sommerfeldt DW, Schmittenbecher P (2021) Failure analysis and recommendations for treatment of posttraumatic non-unions of the distal humerus during childhood. Eur J Trauma Emerg Surg 47, 313–324. 33620527 10.1007/s00068-021-01613-3 PMC8016816

[11] Ranjan R, Sinha A, Asif N, et al. (2018) Management of neglected lateral condyle fracture of humerus. Indian J Orthop 52, 423–429. 30078903 10.4103/ortho.IJOrtho_319_16 PMC6055469

[12] Liu X, Xie L-W, Deng Z-Q, et al (2020) The effect of staged surgical treatment for cubitus valgus after non-union of lateral condylar fracture of distal humerus in older children. Preprint (Version 2) available at Research Square. https://www.researchsquare.com/article/rs-61027/v1.pdf.

